# Exploring natural allies: Survey and identification of larval parasitoids of the American grape berry moth, *Paralobesia viteana* (Lepidoptera: Tortricidae) in northwestern Pennsylvania

**DOI:** 10.1371/journal.pone.0317274

**Published:** 2025-05-19

**Authors:** Jesus H. Gomez-Llano, Neetu Khanal, Flor E. Acevedo

**Affiliations:** 1 Universidade Federal de Pelotas (UFPEL), Av. Eliseu Maciel – Campus Universitário Capão do Leão. Capão do Leão, Rio Grande do Sul, 96010-610, Brasil; 2 Department of Entomology, The Pennsylvania State University, University Park, Pennsylvania, United States of America.; University of Carthage, TUNISIA

## Abstract

The American grape berry moth (GBM), *Paralobesia viteana* (Clemens) (Lepidoptera: Tortricidae) is an economically important pest of grapes. The larvae of this insect burrow inside the fruit upon hatching, consuming, and contaminating grapes and clusters. Current GBM management relies on pesticide applications, which do not offer complete protection due to the cryptic behavior of the larvae and asynchrony in egg-laying, highlighting the need to develop new management strategies. In this study, we identified GBM larval parasitoids in commercial vineyards and quantified their parasitism rates. Parasitoid samplings were conducted biweekly in six conventionally managed ‘Concord’ vineyards in Erie County, Pennsylvania, during the 2023 and 2024 growing seasons. GBM-infested samples were monitored daily to track the emergence of both parasitoids and GBM, enabling the calculation of parasitism rates. We identified eight parasitoid species: *Enytus obliteratus* (Cresson) (Hymenoptera: Ichneumonidae), *Campoplex tortricidis* (Cushman) (Hymenoptera: Ichneumonidae), *Scambus* spp. Hartig (Hymenoptera: Ichneumonidae), *Glypta* cf*. depressa* Dasch, *Glypta* cf. *ohioensis* Dasch, and *Glypta* cf. *ignota* Dasch (Hymenoptera: Ichneumonidae); *Bracon variabilis* (Provancher) (Hymenoptera: Braconidae), and *Goniozus fratellus* Evans (Hymenoptera: Bethylidae) preying on GBM larvae. From these, *B. variabilis*, *E. obliteratus*, and *G. fratellus* were the most abundant. We also designed a graphic taxonomic key to facilitate the identification of these species. The parasitoid abundance differed over the growing season but was greatest in early August, reaching parasitism rates of up to 39% and 52.1% in 2023 and 2024, respectively. Our results demonstrate that GBM has several larval parasitoids that help reduce its populations in commercial vineyards. This research represents a first step toward our understanding of the GBM native natural enemies present in the Lake Erie Region and their potential use in pest management programs.

## Introduction

The grape berry moth (GBM), *Paralobesia viteana* (Clemens) (Lepidoptera: Tortricidae) is a highly destructive pest of grapes in eastern North America [1]. The larvae of this insect feed on grape clusters, causing mechanical injury and increasing the vulnerability to pathogen infection, ultimately, resulting in yield losses and decreased juice and wine quality. Management of GBM currently relies on insecticide sprays timed using a degree-day model developed by Tobin et al. [2]. Although insecticides confer some protection against this insect, their constant use poses human and environmental risks and can lead to the development of GBM resistance to the chemical active substances used [3]. With the long-term goal of developing more sustainable strategies for GBM control, this study focused on the identification of larval parasitoids in ‘Concord’ (*Vitis labrusca* L*.* or *Vitis x labruscana* L.H. Bailey) vineyards in northwestern Pennsylvania (U.S.A).

GBM is native to eastern North America and has coevolved with numerous natural enemies in its original habitat. Studies in the Finger Lakes area (New York), the Lake Erie Region (New York and Pennsylvania), and Michigan have identified several egg and larval parasitoids of GBM (Table 1). However, these studies date back several decades, underlining the need for new surveys to identify current GBM natural enemies that have endured increases in farming land, recurrent pesticide sprays, and climate changes in grape-growing regions. In a 2-year survey conducted in the Finger Lakes region in 1986–87, the natural community of egg and larval GBM parasitoids caused 12–42% mortality throughout the growing season [4]. The most abundant parasitoid in this study was *Trichogramma pretiosum* Riley (Hymenoptera: Trichogrammatidae) to which a large percentage of GBM mortality (20.2%) was attributed. Studies conducted in Michigan vineyards reported *Sinophorus* Förster (Hymenoptera: Ichneumonidae) as the most abundant parasitoid causing 11–76% GBM mortality in field conditions [5]. Surveys in Arkansas vineyards found GBM parasitism of 3.6–48.6% from ichneumonid and braconid parasitoids with no detail of the species reported [6]. Although some work has been conducted in the identification of GBM natural enemies, their efficacy in reducing insect infestations in field conditions remains untested except for one egg parasitoid species, *Trichogramma minutum* Riley. This is a native parasitoid that was found infesting GBM eggs in northwestern Pennsylvania, but its natural parasitism rates were very low to decrease pest populations [7]. Inundative releases of *T. minutum* in commercial vineyards showed a significant decrease in GBM damage [8], demonstrating the potential of natural enemies for controlling this pest.

**Table 1:** Grape berry moth parasitoids previously identified in U.S. vineyards.

Parasitoid species and authority	Order and family	GBM stage they prey on	Reference
*Trichogramma minutum* Riley	Hymenoptera: Trichogrammatidae	Eggs	[7]
*Trichogramma pretiosum* Riley	Hymenoptera: Trichogrammatidae	Eggs	[4]
*Bracon scrutalor* Say	Hymenoptera: Braconidae	Larvae	[17]
*Bathymetis* sp. near *terminalis* Ashmead		Larvae	
*Glypta animosa* Cresson	Hymenoptera: Ichneumonidae	Larvae	
*Glypta vulgaris* Cresson	Hymenoptera: Ichneumonidae	Larvae	
*Thymaris slingerlandana* Ashmead	Hymenoptera: Ichneumonidae	Larvae	
*Glypta mutica* Cushman	Hymenoptera: Ichneumonidae	Larvae	[4,5,18]
*Campoplex tortricidis* (Cushman)	Hymenoptera: Ichneumonidae	Larvae	[18]
*Macrocentrus ancylivorous* Rohwer	Hymenoptera: Braconidae	Larvae	
*Bracon variabilis* (Provancher)	Hymenoptera: Braconidae	Larvae	[4,5]
*Apanteles polychrosidis* Viereck	Hymenoptera: Braconidae	Larvae	
*Elechertus argissa* (Walker)	Hymenoptera: Eulophidae	Larvae	[4]
*Sinophorus* Förster	Hymenoptera: Ichneumonidae	Larvae	[5]
*Enytus obliteratus* (Cresson)	Hymenoptera: Ichneumonidae	Larvae	
*Xorides calidus* (Provancher)	Hymenoptera: Ichneumonidae	Larvae	
*Goniozus foveolatus* Ashmead	Hymenoptera: Bethylidae	Larvae	
*Scambus brevicornis* (Gravenhorst)	Hymenoptera: Ichneumonidae	Larvae	
*Scambus hispae* (Harris)	Hymenoptera: Ichneumonidae	Larvae	
*Bassus annulipes* (Cresson)	Hymenoptera: Braconidae	Larvae	

The biology and ecology of GBM make the control of this insect particularly challenging. GBM evolved with wild grape hosts and became a pest in vineyards established east of the Mississippi River [1]. Adults typically emerge from overwintered pupae between May and June [9,10]. However, the termination of diapause is variable, lasting about six weeks [10], resulting in asynchronous egg laying over the growing season. Mated females oviposit on developing buds, flower clusters, or berries from wild and cultivated grapevines [1,11]. Females lay an average of 60 eggs in their lifetime, from which more than 86% successfully hatch in controlled conditions [12]. Larvae emerge after 3–4 days and create webs in the clusters [1,9]. Early in the season, larvae feed on buds or flower clusters, whereas later in the season, they burrow into the fruit within hours of egg-hatch. Inside the grape berries, larvae remain protected against chemical control while consuming the internal tissue of the berries. The larval period takes an average of 18.5 days at 25 ± 1 °C [12]. After fully developed, fourth instar larvae exit the berry and pupate by cutting a flap in a grapevine leaf and forming a silken chamber [9,10]. The pupa stage takes an average of 9.52 days to develop at 25 ± 1 °C, and the longevity of adults is on average 15.6 days in controlled conditions [12]. The oviposition period is 7.3 days on average, with some variation associated with the grape cultivar [12]. The number of GBM generations per season varies with temperature accumulation; there are two to three generations in the Lake Erie region and central New York State [1,13–15], whereas, in southern Missouri and Arkansas, there can be up to four generations [1,16]. In the Lake Erie region, the first GBM generation develops in wild grapes (*Vitis* spp. L), and subsequent generations develop in cultivated grapes, where they cause significant crop losses [7]. Access to widespread wild hosts, the cryptic feeding habit of the larvae, the prolonged oviposition period of females, and the large variation of diapause termination contribute to the challenges of controlling this insect.

The variation in diapause termination results in constant GMB pressure that reduces the effectiveness of chemical control that targets peaks of egg-laying [10]. Therefore, a long-lasting control strategy that reduces pest populations over several weeks would be ideal. Biological control using egg and larval parasitoids holds great potential for controlling GBM because they may have the capability of controlling the pest for several days. Additionally, parasitoids may be able to control GBM in both wild and cultivated grapes. Augmentative releases of parasitoids have great potential to reduce GBM populations below economic injury levels [7]. Furthermore, parasitoids could offer long-lasting pest control if they successfully establish in the field, but research is needed to determine the feasibility of this strategy.

In this study, we surveyed and assessed the parasitism rates of GBM larval parasitoids in six vineyards in Erie County (Pennsylvania) throughout the 2023 and 2024 growing seasons. We also developed a graphical taxonomic key to facilitate further identification of these species. Our results provide knowledge of the current diversity of GBM larval parasitoids in commercial vineyards of northwestern Pennsylvania and would be a practical basis for future studies to test the effectiveness of the identified species in controlling GBM in field conditions.

## Materials and methods

### Sampling sites and specimen collection from the field

Surveys were conducted biweekly from June to September 2023 and 2024 in six privately-owned conventional vineyards located in Erie County, northwestern Pennsylvania. Among these six sites, four (Site 1: 42°11’21.0“N, 79°52’58.6”W; Site 2: 42°12’29.0”N, 79°53’26.6”W; Site 3: 42°12’30.9”N, 79°53’31.7”W, and Site 4: 42°13’30.1”N, 79°49’10.5”W) were situated in North East, Pennsylvania, to the east of Erie city, while the remaining two sites (Site 5: 42°02’03.6”N, 80°18’23.8”W, and Site 6: 42°01’54.0”N, 80°17’20.8”W) were located to the west of Erie city. These vineyards were selected for their recurrent history of GBM infestation and were all planted with the grape cultivar ‘Concord’. These vineyards also had at least one border near a deciduous wood area and received insecticide applications according to each grower’s standard insect control program. The size of the vineyards and adjacent wooded areas varied at each sampling site (see S1 Table). Grapevine blossoms and grapes with signs of GBM infestation (blossoms or grapes attached with silk and purple-colored grapes with holes) were collected from the six sites throughout the growing season in both years. All samples were collected from vineyard borders as previous research indicates that borders are more heavily infested with GBM and, consequently, have a greater number of natural enemies [4,5]. The first sampling in 2023 (June 15) was from wild grapevines growing at the borders of the cultivated plots and the subsequent samples were obtained from the adjacent commercial ‘Concord’ vineyards. During the first sampling, we collected several bunches of wild grape blossoms, while in the subsequent samplings, we collected ~ 100–150 grapes per site from the vineyard borders adjacent to wooded areas. In 2024, we attempted to collect samples from wild grapes in early June, but there were too few insects from only a few sites. Therefore, all our samplings for this year were from commercial vineyards from June 24 to September 2^nd^.

### Specimen rearing in laboratory conditions and calculation of parasitism rates

Field-collected specimens were taken to a laboratory located in Penn State Behrend (Erie, Pennsylvania) for rearing and collection of GBM larval parasitoids. The samples were kept in a Conviron growth chamber (GEN200SH) at a temperature of 25 ± 1 °C, 70% ± 2% relative humidity (RH), and a photoperiod of 16:8 h (L:D). Grape blossoms from wild grapes were inspected under a stereoscope (Leica S9D Nuhsbaum Inc, McHenry Il, USA) to carefully remove GBM larvae using a soft fine paintbrush; each larva was individualized in a 1 oz plastic cup (Dart Conex Complements 301100PC, WebstaurantStore, Landcaster Pennsylvania) and fed with wild grapes collected from the field. Each cup was provided with a piece of paper towel (~2 squared cm) as a substrate for GBM pupation. The cups were inspected periodically to record moth or parasitoid emergence. GBM-infested grapes were also taken to the lab and placed in 450 ml plastic containers (6 cm tall, 11 cm in diameter) covered at the top with a fine fabric mesh fixed with a rubber band. Pieces of paper towel (~2 squared cm) were placed inside the containers as substrate for GBM pupation. The cups were periodically inspected for GBM or parasitoid pupae as well as for adult emergence. The number of GBM and the number of parasitoids were recorded for each site and sampling time.

### Parasitoid identification and development of a taxonomic key

Adult parasitoid specimens were stored in 1.5 ml plastic tubes either dry at -20°C or in 70% ethanol. These specimens were identified at the genus and species level using taxonomical keys for the families Ichneumonidae [19–22], Bethylidae [23], and Braconidae [24,25]. In addition, entomological material from the Frost Entomological Museum at Penn State University (State College, Pennsylvania), the Cornell University Insect Collection (Ithaca, NY), and the USDA Systematic Entomology Laboratory (Smithsonian Institution, Washington D.C.) were reviewed to confirm the taxonomic identification of the specimens. Voucher specimens are stored in the Frost Entomological Museum, and extra samples are kept at Flor Acevedo’s laboratory. Lastly, we developed a taxonomic key following the terminology proposed by Townes [19], Evans [23], and Achterberg [24], to facilitate the identification of the primary parasitoids documented in this study. Images of parasitoid specimens were taken under a Leica S9D stereomicroscope with an attached digital camera Canon EOS 90D (Melville, NY). Images of small specimens and body structures were taken with an FEI quanta 650 FEG Environmental Scanning Electron microscope (SEM).

### Data analyses

We calculated the percent of parasitism in the samples collected from the field for each sampling site and sampling date in 2023 and 2024. The parasitism rates were expressed as the number of parasitoids divided by the sum of parasitoids and GBM that emerged multiplied by 100. The effects of the different sites and sampling times on the mean percent of parasitism were assessed separately for each year using a beta regression model with a logit link function for the mean (*mu*) and an identity link function for the dispersion parameter (*phi*) using the response variable as a proportion between 0 and 1. We used *phi* coefficients to determine the variability of the data and log-likelihood to determine how well the model fitted the data [26]. With these methods, we tested the null hypothesis (H_0_) of no differences in the percent of parasitism between sites and sampling times.

The parasitoid community composition in the sampling sites for each year was assessed by calculating the diversity indexes of Shannon-Weaver and Simpson using the “vegan” R package [27]. Statistical differences in the Shannon-Weaver diversity indexes between sites were assessed with Hutcheson’s t-test [28]. All statistical analyses and graphs were made in R [29].

### Ethics statement

A formal institutional permit for collecting the insect specimens was not required for this study because they are not considered endangered species, and they were collected from private land. Oral permission for conducting insect samplings was obtained from landowners during the duration of the study. Written consent was not deemed necessary because this study was not classified as human subjects’ research. The information disclosed in this study does not pose any risk to individuals or their privacy, as far as the authors are aware.

## Results

### Field parasitism rates

**Fig 1:**
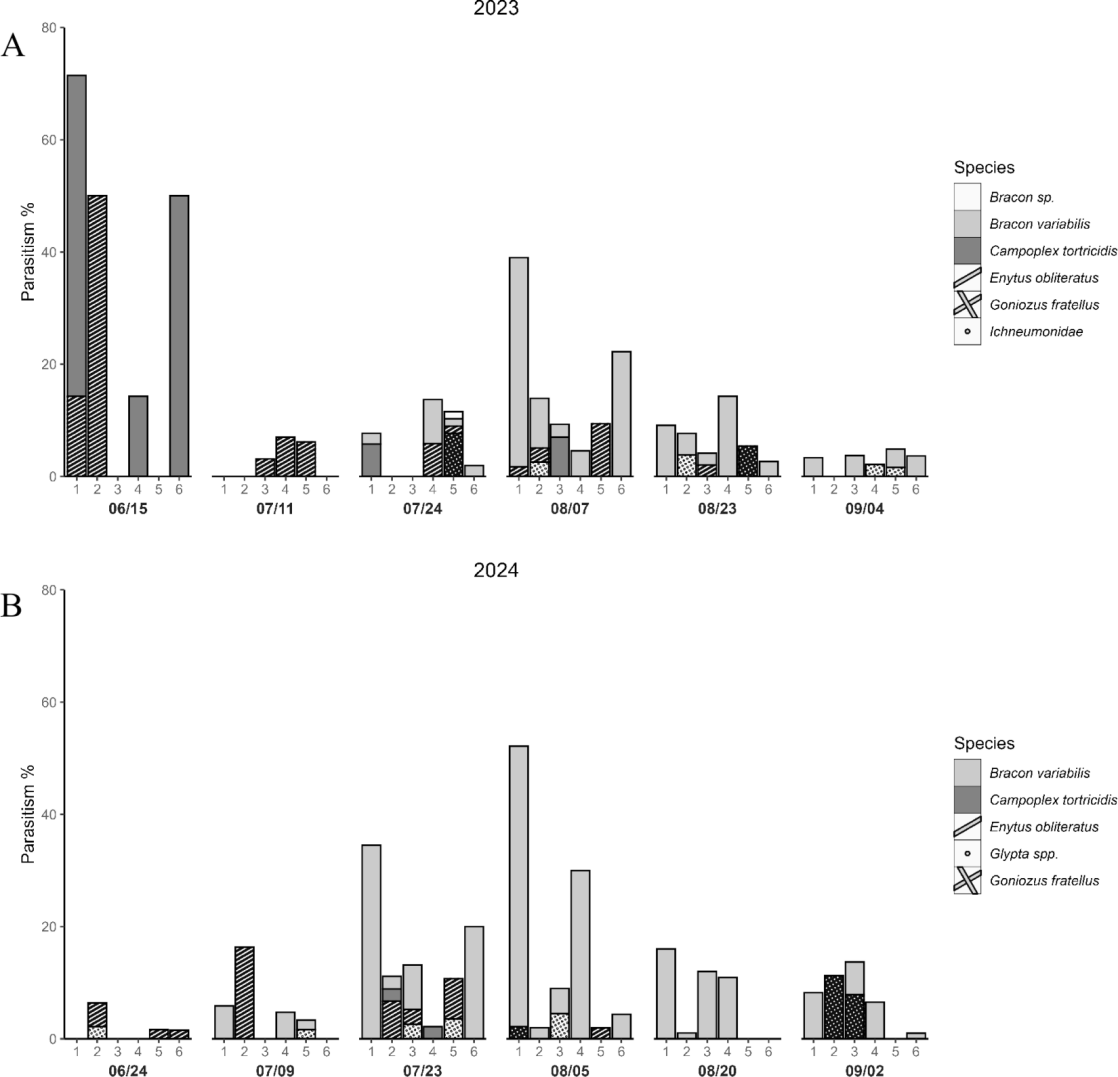
Field parasitism rates of GBM larval parasitoids. Percent of parasitism of GBM larval parasitoids in six different sites (1–6) and sampling times over the growing seasons of **(A)** 2023 and **(B)** 2024.

We collected a total of 113 and 159 GBM larval parasitoid specimens from all sites and samplings carried out in 2023 and 2024, respectively. In 2023, the highest parasitism rates were observed in the first sampling of the season in wild grapes (30.95% on average; *Z* = -5.4, *P* < 0.001), however, the number of samples with GBM living stages was very few in this sampling. The second highest parasitism rates in 2023 were found in the sampling of August 7 with an average of 16.4% across sites, while the lowest was found on July 11 with 2.7%. In 2023, site 1 had the highest parasitism across sampling dates with an average of 21.7% (*Z* = -3.4, *P* < 0.001), followed by site 6 with 13.6% and site 2 with 12%. In the same year, site 3 had the lowest parasitism rates with an average of 3.4% (Fig 1A; S2 Table). In 2024, the percentage of parasitism between sampling times was not statistically different (*P* > 0.05). Descriptively, the average highest parasitism rate across sites was 16.4% in the sampling conducted on August 5^th^, followed by the sampling conducted on July 23 (15.27%) in 2024. The average lowest parasitism rates in this year were found on June 24, with 1.6%. Site 1 had the highest parasitism across sampling dates, with an average of 19.3% (*Z* = -4.2, *P* < 0.001 followed by site 4, with 9%, and site 2, with 8% in 2024. In this year, site 5 had the lowest parasitism rate with 3% (*Z* = -2.37, *P* < 0.05; Fig 1B; S3 Table).

### Parasitoid identification and abundance

**Fig 2:**
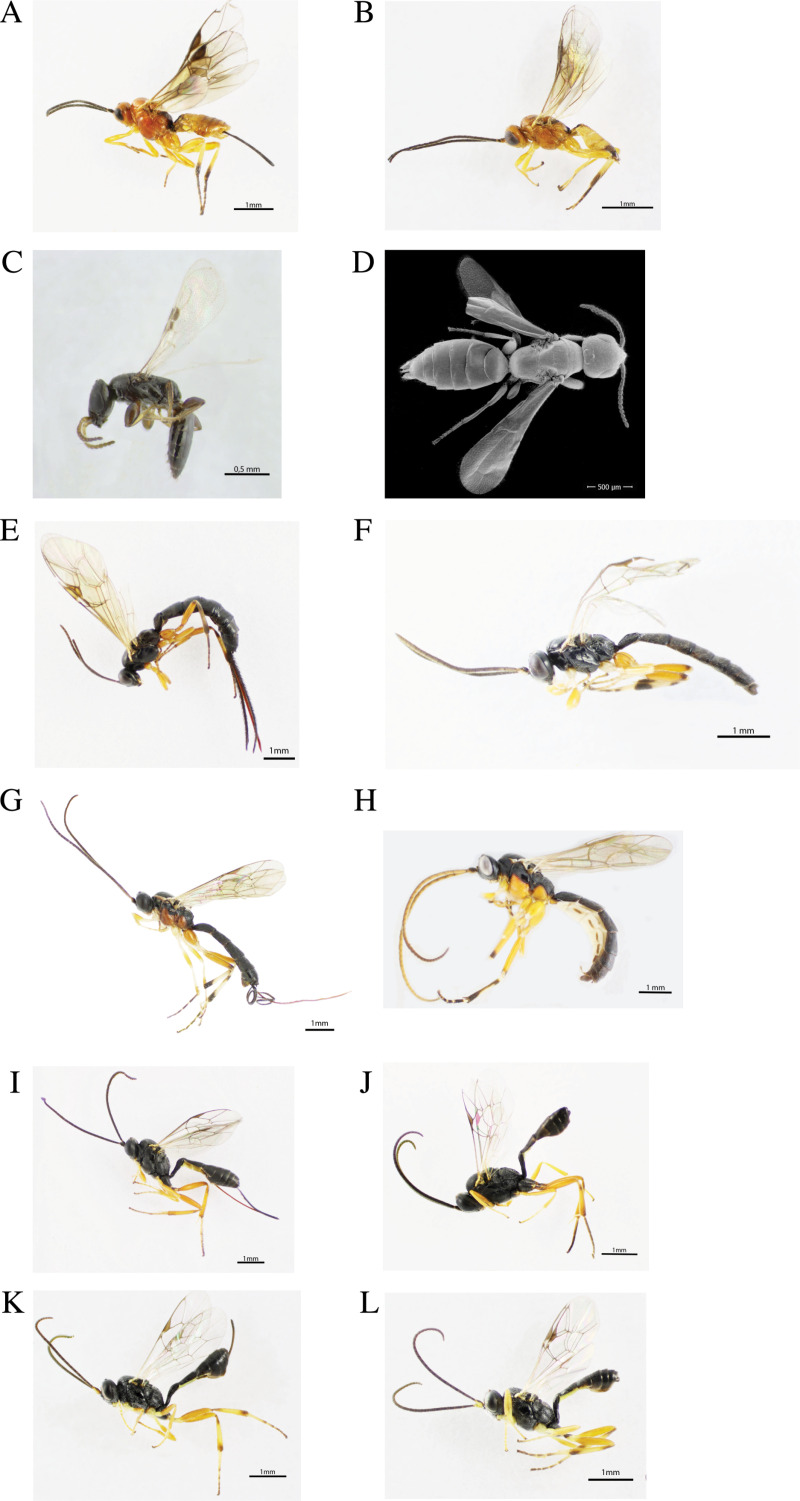
Grape berry moth larval parasitoids identified in northwestern Pennsylvania. **(A-B)** *Bracon variabilis*, **(C-D)** *Goniozus fratellus,* **(E-F)** *Scambus* spp., **(G-H)** *Glypta* spp., **(I-J)** *Campoplex tortricidis*, **(K-L)** *Enytus obliteratus.*

In our survey, we identified a total of eight parasitoid species belonging to three Hymenoptera families. The identified species were *Enytus obliteratus* (Cresson) (Hymenoptera: Ichneumonidae) [30], *Campoplex tortricidis* (Cushman) (Hymenoptera: Ichneumonidae) [20]*, Scambus* spp. Hartig (Hymenoptera: Ichneumonidae) [31], three possible species of the genus *Glypta* (Hymenoptera: Ichneumonidae): *Glypta* cf. *depressa* Dasch [21], *Glypta* cf. *ohioensis* Dasch [21], and *Glypta* cf. *ignota* Dasch [21]. *Bracon variabilis* (Provancher) (Hymenoptera: Braconidae) [32]*,* and *Goniozus fratellus* Evans (Hymenoptera: Bethylidae) [23]. Additionally, we found three morphospecies within the *Scambus* genus, and one within *Glypta* that could not be identified further due to the lack of taxonomic keys. Since additional research is needed to confirm the *Glypta* and *Scambus* species, we grouped all these specimens within their respective genera (i.e., *Glypta* spp. and *Scambus* spp.) for further analyses and graphs. From the specimens found, *B. variabilis* and *G. fratellus* are ectoparasitoids, and the others are endoparasitoids. We collected the same parasitoid species in 2023 and 2024, except for *Scambus* spp., which was absent in our 2024 samplings. The most abundant parasitoid species are depicted in Fig 2.

The abundance of these parasitoids in the field varied throughout the growing season. In 2023, the most abundant parasitoid species were *B. variabilis* and *E. obliteratus*, comprising 56.6% and 18.6% of all parasitoids found, respectively (Fig 1A). In 2024, *B. variabilis* was also the most abundant species, representing 68.6% followed by *G. fratellus* and *E. obliteratus* comprising 13.8% and 11.9%, respectively, of all parasitoids found (Fig 1B). *Bracon variabilis* was recovered from samples collected from July to September, whereas *E. obliteratus* was only present from June to mid-August. Notably, *G. fratellus* was only found late in the season (from late July to September). The remaining species were randomly found in samples collected throughout the season (Fig 1).

**Fig 3:**
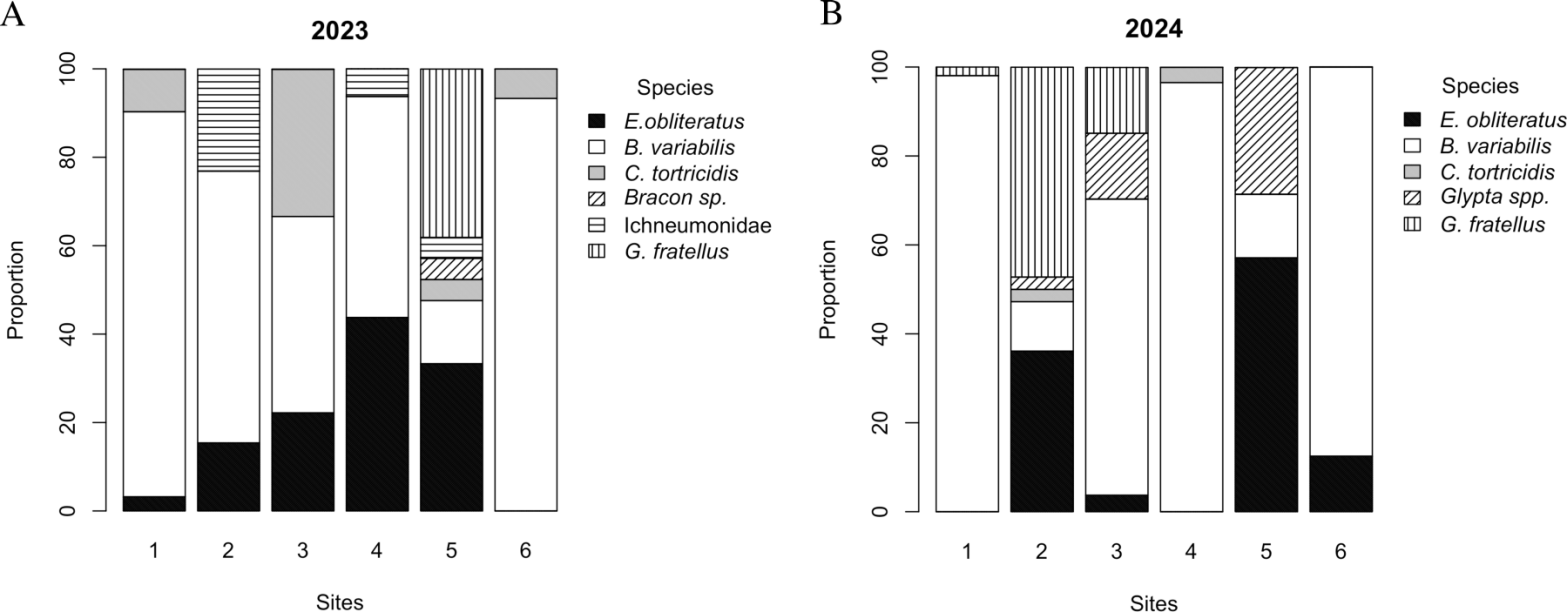
Abundance of GBM larval parasitoids across six different sites in (A) 2023 and (B) 2024.

There were also differences in the parasitoid community composition in the sampling sites. In 2023, site 5 had the highest diversity compared with sites 1 and 6 (*P* < 0.001), but was no different from sites 2, 3, and 4 (*P* > 0.05) (Table 2, Fig 3A). In 2024, sites 2, 3, and 5 had the highest diversity compared with sites 1 and 4 (*P* < 0.001). Site 2 was also different from site 6 (*P* < 0.005) (Table 2, Fig 3B). In general, sites 2 and 5 were the most diverse during the two years of the study (Table 2, Fig 3).

**Table 2:** Alpha diversity indexes for each vineyard site (1–6) during 2023 and 2024.

Site	2023			2024		
**Shannon (H)**	**Simpson**	**Richness**	**Shannon (H)**	**Simpson**	**Richness**
1	0.4571	0.231	3	0.095	0.0377	2
2	0.9251	0.5443	3	1.1653	0.6327	5
3	1.0609	0.6419	3	0.9582	0.51	4
4	0.8815	0.5548	3	0.15	0.06658	2
5	1.4468	0.7165	6	0.9557	0.5714	3
6	0.2449	0.1244	2	0.3768	0.2187	2

### Taxonomic key for the GBM parasitoids identified in this study

**Fig 4:**
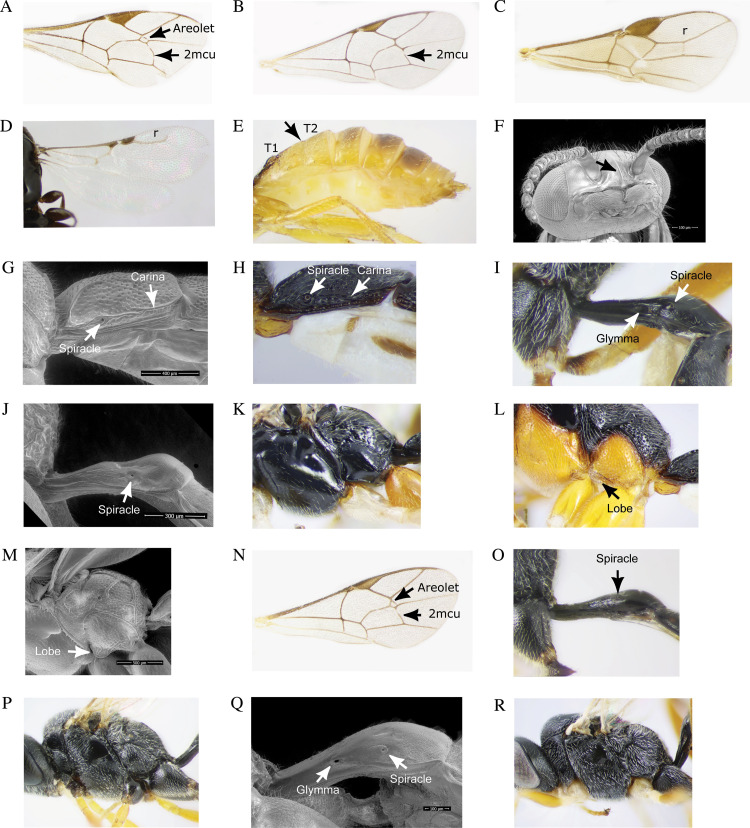
Key morphological characters for the identification of the GBM larval parasitoids found in northwestern Pennsylvania. **(A)** subtriangular areolet and vein 2m-cu, **(B)** absent areolet with vein 2m-cu present, **(C)** enclosed radial cell, **(D)** open radial cell, **(E)** 2^nd^ and 3^nd^ tergites fused, **(F)** clypeus with a strong angular or subangular median lobe, **(G-H)** spiracle of 1^st^ tergite with a complete dorsolateral carina, **(I and Q)** spiracle of the first abdominal tergite placed posteriorly and glymma, **(J and O)** spiracle of first abdominal tergite with absent glymma, **(K)** carina forming a lobe, **(L-M)** submetapleura carina extending anteriorly forming a lobe, **(N)** closed areolet before the junction with radius, **(P)** black coxae, **(R)** yellow fore and mid coxae.

1a Fore wing vein 2m-cu present (Figs 4A–B) …………............................................................. 3
1b Fore wing vein 2m-cu absent; hind wing venation reduced (Figs 4C–D).................................2
2a Face and clypeus separated by a groove, mandibles sometimes exodont; 2^nd^ and 3^nd^ tergites usually fused (Fig 4E); predominantly yellow coloration; radial cell completely enclosed (Fig 4C)…………………………...………………...…….………...…….Braconidae (*Bracon variabilis;* Figs 2A–B).
2b Not agreeing with the above; clypeus with a strongly produced angular or subangular median lobe (Fig 4F); predominantly black coloration; radial cell not enclosed (Fig 4D)...................................................................................………...……..Bethylidae (*Goniozus fratellus;* Figs 2C–D).
3a Spiracle of 1^st^ tergite placed behind or around the middle of the tergite (Figs 4G–H) ……….4
3b Spiracle of 1^st^ tergite placed at posterior third of the tergite (Figs 4I and Q) …….……..……5
4a Areolet present and subtriangular, wider than high (Fig 4A); nervellus intercepted below the middle or in some cases in the middle; submetapleura carina not extending anteriorly forming a lobe (Fig 4K)..................................................................... Ichneumonidae (*Scambus*; Figs 2E–F).
4b Areolet absent (Fig 4B); nervellus intercepted near hind end the middle; dorsolateral carina of the first tergite complete (Fig 4G); strong submetapleura carina extending anteriorly forming a lobe (Figs 4L–M)...................................................................Ichneumonidae (*Glypta*; Figs 2G–H).
5a Areolet present and closed before the junction with radius (Fig 4N); glymma absent (Figs 4J and O); all coxae black (Fig 4P)*......................*Ichneumonidae (*Campoplex tortricidis*; Figs 2I–J).
5b Areolet absent (Fig 4B); glymma present (Figs 4I–Q); fore and mid coxae yellow (Fig 4R) ….......................................................................Ichneumonidae (*Enytus obliteratus*.; Figs 2K–L).

## Discussion

The GBM, *P. viteana* has an abundant number of larval parasitoids that provide significant natural control. We successfully identified eight hymenopteran parasitoids in samples from six commercial ‘Concord’ vineyards in northwestern Pennsylvania over two consecutive years (2023 and 2024). These vineyards were conventionally managed following current spray recommendations [33], indicating that natural control still occurs despite insecticide use. Field parasitism rates varied through the growing season and ranged from 0 to 39% in 2023 and from 0 to 52.1% in 2024. This study represents a first step toward our understanding of the native natural enemies present in the Lake Erie Region and their use in pest management programs.

The community of larval parasitoids identified in this study confirms previous species records and provides new findings. *E. obliteratus*, *C. tortricidis, B. variabilis,* and *Scambus* spp. have been previously reported parasitizing *P. viteana* [4,5,18], but *G. fratellus, G.* cf*. depressa*, *G.* cf*. ohioensis*, and *G.* cf*. ignota* are new reports for this species. In our samplings, the most abundant parasitoid species was *B. variabilis* accounting for 56.6% (in 2023) and 68.6% (in 2024) of all parasitoids found. However, in similar studies conducted in 1986–87 in the Finger Lakes region in New York, the most abundant species was the egg parasitoid *T. pretiosum*, followed by the larval parasitoids *A. polychrosidis,* and *G. mutica* from the eight species found [4]. In a 3-year (2003–2005) study in Michigan vineyards, *Sinophorus* sp. was the most abundant parasitoid, comprising 11–76% of all the specimens collected from 12 identified species [5]. These results confirm the presence of a large number of GBM parasitoids in the eastern U.S. and suggest that the community composition and abundance of these species vary in different grape-growing regions.

The field parasitism rates fluctuated over the growing season and habitat. In 2023, the highest percent of parasitism was from samples collected in wild grapes early in the season and ranged from 0 to 71.4% (Fig 1A; S2 Table). Similar results were found by Seaman et al. [4], who reported higher parasitism in wild grapes reaching up to 65%. The highest parasitism rates in cultivated grapes were found during the first week of August and ranged from 4.5 to 22.2% in 2023 and from 2 to 52.1% in 2024 (Fig 1; S2 and S3 Tables). In contrast, the lowest percentages of parasitism were found during the second week of July 2023 and the last week of June 2024. Similarly, Jenkins et al. [34] found a low abundance of parasitoids early in the season that increased in mid-August to levels of up to 30% and declined as the season progressed. In the Lake Erie region, the first generation of GBM emerges before the cultivated grapes bloom and feeds on developing blossoms from wild grapes. In comparison to later generations that burrow into grape berries, these larvae are highly exposed to parasitoids and predators despite building protective silk enclosures. Gleisner [18] suggested that berries may protect the larva from parasitoids with short ovipositors. The variation in parasitism through the season could be associated with varying availability of appropriate GBM instars for parasitoids to use, variation in GBM infestation levels, or asynchrony between parasites and the host [5,18]. Other possibilities include changes in the availability of alternate hosts for parasitoids or differences in the timing of diapause initiation for the parasitoids and the host later in the season.

All the GBM parasitoids found in this study have alternate hosts. The most abundant parasitoid species in our samplings was the ectoparasitoid *B. variabilis,* whose parasitism rates ranged from 1.3–37.3% in 2023 and from 1.6–51.1% in 2024. This species was previously reported parasitizing GBM in Van Buren and Berrien Counties in Michigan and in the Finger Lakes region in NY [4,5]. Other lepidopteran hosts of *B. variabilis*, include the nantucket pine tip moth, *Rhyacionia frustrana* (Scudder) (Lepidoptera: Tortricidae) in Georgia [35], the hickory shuckworm moth, *Cydia caryana* (Fitch) (Lepidoptera: Tortricidae) in Texas [36], and the alder tubemaker moth, *Acrobasis rubrifasciella* Packard (Lepidoptera: Pyralidae) in the Eagles Nest Lakes region, near Ely, Minnesota [37]. *Bracon variabilis* has also been reported parasitizing the curculionid flower beetle, *Baris transversa* (Say & T.) [38]. The endoparasitoid *E. obliteratus* was the second most abundant species with parasitism rates in commercial vineyards ranging from 1.3 to 9.4% in 2023 and from 1.5 to 16.3% in 2024. This species has been previously reported parasitizing GBM in Michigan State [5]. *Enytus obliteratus* also parasitizes larvae of the European GBM, *Lobesia botrana* (Denis & Schiffermüller) [39], and larvae of the oriental fruit moth, *Grapholita molesta* (Busck), an important pest of stone fruits (peaches, cherries, etc). It has also been reported as a parasitoid of *Arogalea cristifasciella* (Chambers) (Lepidoptera: Gelechiidae) larvae [40]. This genus has primarily a Nearctic and Palearctic distribution [41]. The ectoparasitoid *G. fratellus* had parasitism rates that fluctuated from 5.4 to 7.7% in 2023 and from 1 to 11.2% in 2024. To our knowledge, this is the first time this species has been reported preying on GBM. *G. fratellus* also parasitizes *Acrobasis nuxvorella* Neunzig (Lepidoptera: Pyralidae) [36]. This species has been reported in several States, including California, Nevada, Arizona, New Mexico, Georgia, Texas, Florida, South Carolina, Michigan, Connecticut, Massachusetts, Ontario, and Louisiana [23], without information about the hosts. The endoparasitoid *C. tortricidis* had parasitism rates below 2%. This species was first found by Cushman [20] in North East, Pennsylvania, parasitizing *P. viteana,* and was reported again by Gleissner [18] from GBM samples gathered between 1940–1943 in the same geographic location. It was also reported parasitizing the oriental fruit moth in Ohio [42]. Information on this species is scarce and the only other geographical record besides northwestern Pennsylvania is the one in Edmonton, Canada [43,44].

There were also three species of *Glypta* identified in this study: *G.* cf*. depressa*, *G.* cf*. ohioensis*, and *G.* cf*. ignota,* which together had field parasitism rates below 5%. To our knowledge, this is the first report of these species parasitizing GBM and no other hosts have been reported in the literature. Information on their geographic distribution is also limited. *G.* cf*. depressa* has been recorded in New Mexico, whereas *G.* cf*. ohioensis* has been found in Ohio, Louisiana, Michigan, New York, Pennsylvania, South Carolina, West Virginia and Ontario [21]. For *G.* cf*. ignota*, there is only one report in Nova Scotia [21]. Other species of this genus, such as *G. animosa*, *G. vulgaris*, and *G. mutica* are known parasitoids of *P. viteana* [5,17]. Natural parasitism rates of *G. mutica* in the Finger Lakes region ranged from 0.01 to 6.4% and in caged experiments varied from 3 to 7% [4]. *Glypta* species in general, are known parasites of several Lepidoptera families, including Cochylidae, Gelechiidae, Geometridae, Lasiocampidae, Lycaenidae, Lymantriidae, Momphidae, Noctuidae, Pyralidae and Tortricidae. This genus is found principally in the Holarctic and is less frequent in the Neotropical and Oriental regions.

*Scambus* spp*.* was a less frequent parasitoid found in our study only in the 2023 samplings, with parasitism lower than 1%. Two species within this genus have been previously identified parasitizing *P. viteana*: *S. brevicornis* and *S. hispae* in Michigan vineyards [5]. However, other *Scambus* species have been reported parasitizing other tortricids. For example, *Scambus elegans* (Woldstedt) parasitize *L. botrana* [45], whereas *Scambus calobatus* (Gravenhorst) and *Scambus vesicarius* (Ratzeburg) parasitize the European grapevine moth, *Eupoecilia ambiguella* (Hübner) (Lepidoptera: Tortricidae) [46–48]. There are also reports of *Scambus* species parasitizing Diptera and Coleoptera insects [49–51]. This genus is widely distributed in the Northern Hemisphere [19].

The abundance of the GBM parasitoids found in this study fluctuated over the course of the growing season. *E. obliteratus, C. tortricidis* and *Glypta* spp*.* were only present from June to mid-August, whereas *B. variabilis* and *G. fratellus* were found from late July to September (Fig 1). This temporal variation could be associated with different thermal requirements and diapause periods for the different species and could be helpful in reducing interspecific competition of parasitoids for the same prey.

We conclude that the American GBM, *P. viteana* is host for several larval parasitoids in commercial ‘Concord’ vineyards in Pennsylvania. We confirmed previous species reports and added four new records to the parasitoid list of this species. The parasitoid abundance differed over the season but was greatest in early August, reaching parasitism rates of up to 52.1%. Despite their importance, information about these parasitoids is limited; future research is needed to document the biology and ecology of the most promising species to identify ways to increase their natural populations in vineyards. Measurements should be taken to protect the community of natural enemies that prey on GBM in vineyards. Some strategies, include reducing the use of broad-spectrum insecticides, using economic thresholds to determine the need for insecticide applications, and protecting wild vegetation that provides food for parasitoid adults.

## Supporting information

S1 Table Characteristics of the grape berry moth sampling sites. (PDF)

S2 Table GBM larval parasitism in field conditions per sampling site throughout the 2023 growing season. (PDF)

S3 Table GBM larval parasitism in field conditions per sampling site throughout the 2024 growing season. (PDF)

## References

[1] Isaacs R, Teixeira LAF, Jenkins PE, Neerdaels NB, Loeb GM, Saunders MC. Biology and management of grape berry moth in North American vineyard ecosystems. In: Bostanian NJ, Vincent C, Isaacs R, editors. Arthropod management in vineyards: Pests, approaches, and future directions. 1st ed. The Netherlands: Springer Dordrecht; 2012. pp. 361–81. doi: 10.1007/978-94-007-4032-7_15

[2] Tobin PC, Nagarkatti S, Saunders MC. Modeling development in grape berry moth (Lepidoptera: Tortricidae). Environ Entomol. 2001;30(4):692–9. doi: 10.1603/0046-225x-30.4.692

[3] Nagarkatti S, Tobin PC, Muza AJ, Saunders MC. Carbaryl resistance in populations of grape berry moth (Lepidoptera: Tortricidae) in New York and Pennsylvania. J Econ Entomol. 2002;95(5):1027–32. doi: 10.1093/jee/95.5.1027 12403430

[4] Seaman AJ, Nyrop JP, Dennehy TJ. Egg and larval parasitism of the grape berry moth (Lepidoptera: Tortricidae) in three grape habitats in New York. Environ Entomol. 1990;19(3):764–70. doi: 10.1093/ee/19.3.764

[5] Jenkins PE. Control of the grape berry moth, *Paralobesia viteana*, using reduced-risk insecticides, cultural controls, and conservation of natural enemies. M.Sc. Thesis, Michigan State University. 2006. Available from: https://d.lib.msu.edu/etd/33972

[6] Williamson JR, Johnson DT. Effects of grape berry moth management practices and landscape on arthropod diversity in grape vineyards in the Southern United States. horttech. 2005;15(2):232–8. doi: 10.21273/horttech.15.2.0232

[7] Nagarkatti S, Muza AJ, Saunders MC, Tobin PC. Role of the egg parasitoid *Trichogramma minutum* in biological control of the grape berry moth, *Endopiza viteana*. BioControl. 2002; 47: 373–85. doi: 10.1023/A:1015679710995

[8] Nagarkatti S, Tobin PC, Saunders MC, Muza AJ. Release of native *Trichogramma minutum* to control grape berry moth. Can Entomol. 2003;135(4):589–98. doi: 10.4039/n02-099

[9] Luciani MA. The biology of the grape berry moth, *Endopiza viteana* (Clemens) (Lepidoptera: Tortricidae) in southern Ontario. M. Sc. thesis, University of Guelph. 1987.

[10] Tobin PC, Nagarkatti S, Saunders MC. Diapause maintenance and termination in grape berry moth (Lepidoptera: Tortricidae). Environ Entomol. 2002;31(4):708–13. doi: 10.1603/0046-225x-31.4.708

[11] Clark LG, Dennehy TJ. Oviposition behavior of grape berry moth. Entomol Exp Appl. 1988;47(3):223–30. doi: 10.1111/j.1570-7458.1988.tb01140.x

[12] Laiton-Jimenez L, Samiksha F, Acevedo FE. Biology and life table parameters of *Paralobesia viteana* (Lepidoptera: Tortricidae), grown on different grape cultivars. J Econ Entomol. 2024;117(3):1152–63. doi: 10.1093/jee/toae080 38691142

[13] Ingerson HG. Life history of the grape berry moth in northern Ohio. Washington, D.C: United States Department of Agriculture Bull No. 911; 1920.

[14] Gleissner BD, Worthley HN. Evidence for a third brood of the grape berry moth, *Polychrosis viteana* Clemens, in the Great Lakes region. J Econ Entomol. 1941; 34: 426–31. doi: 10.1093/jee/34.3.426

[15] Hoffman CJ, Dennehy TJ, Nyrop JP. Phenology, monitoring, and control decision components of the grape berry moth (Lepidoptera: Tortricidae) risk assessment program in New York. J Econ Entomol. 1992; 85: 2218–27. doi: 10.1093/jee/85.6.2218

[16] Biever KD, Hostetter DL. Phenology and pheromone trap monitoring of the grape berry moth, *Endopiza viteana* Clemens (Lepidoptera: Tortricidae) in Missouri. J Entomol Sci. 1989;24:472–81. doi: 10.18474/0749-8004-24.4.472

[17] Slingerland MV. The grape berry moth. Cornell University Agricultural Experimental Station of the College of Agriculture Bull. 1904;223:43–60.

[18] Gleissner BD. Biology and control of berry moth in the Erie grape belt: With notes on other grape insects. U.S.A: Pennsylvania State College, School of Agriculture, Agricultural Experiment Station Bull; 1943.

[19] Townes H. The genera of ichneumonidae part 1. Ann Arbor, Michigan, U.S.A: The American Entomological Institute. 1969.

[20] Cushman RA. Descriptions of new Ichneumonidae and taxonomic notes. Proc Entomol Soc Wash. 1915;17:132–42.

[21] Dash CE. Ichneumon-flies of America north of Mexico: 10. subfamily Banchinae, tribe Glyptini. Florida, U S A: The American Entomological Institute. 1988.

[22] Townes H, Townes M. Ichneumon-flies of America north of Mexico: part 7. Subfamily Banchinae, tribes Lissonotini and Banchini. Ann Arbor, Michigan, U.S.A: The American Entomological Institute. 1978.

[23] Evans HE. The Bethylidae of America north of Mexico. American Entomological Institute. 1978.

[24] Achterberg C van. Illustrated key to the subfamilies of the Braconidae (Hymenoptera: Ichneumonoidea). Leiden: Zoologische Verhandelingen; 1993.

[25] Achterberg C. Illustrated key to the subfamilies of the Holarctic Braconidae (Hymenoptera: Ichneumonoidea). Zool Med Leiden. 1990;64:1–20.

[26] Ferrari S, Cribari-Neto F. Beta regression for modelling rates and proportions. J Appl Stat. 2004; 31: 799–815. doi: 10.1080/0266476042000214501

[27] Oksanen J. vegan: Community ecology package. R package version 2.6-8; 2024. Available from: https://CRAN.R-project.org/package=vegan

[28] Hutcheson K. A test for comparing diversities based on the Shannon formula. J Theor Biol. 1970;29(1):151–4. doi: 10.1016/0022-5193(70)90124-4 5493290

[29] R core Team. R: A language and environment for statistical computing. R Foundation for Statistical Computing, Vienna, Austria; 2024. Available from: https://www.R-project.org.

[30] Cresson E. Descriptions of North American Hymenoptera in the collection of the entomological society of Philadelphia. Proc Entomol Soc Phila. 1864;3:257–321.

[31] Hartig T. Ueber den raupenfrass im konigl. Charlottenburger forste Unfern Berlin, wahrend des sommers 1837. Jahresber Fortschr Forstwiss Forstl Naturk Berlin. 1838;1:246–74.

[32] Provancher L. Additions a la faune hymenopterologique. Nat Can. 1888;17:273–440.

[33] Brown B, Gold K, Hed BE, Helms M, Loeb G, Sosnoskie L. 2024 New York and Pennsylvania pest management guidelines for grapes. Ithaca, New York: Cornell Cooperative Extension Pesticide Safety Education Program (CCE-PSEP); 2024. Available from: https://www.cornellstore.com/2024-pmep-guide-for-ny-and-pa-grape-mgmt?location=&quantity=1&size=85

[34] Jenkins PE, Isaacs R. Reduced-risk insecticides for control of grape berry moth (Lepidoptera: Tortricidae) and conservation of natural enemies. J Econ Entomol. 2007;100:855–65. doi: 10.1093/jee/100.3.855 17598548

[35] Wells ML, Hagan DV, McPherson RM. Survey of parasitoids associated with *Rhyacionia frustrana* (Comstock) in Bulloch County, Georgia. J Entomol Sci. 2001;36:101–4. doi: 10.18474/0749-8004-36.1.101

[36] Gunasena GH, Harris MK. Parasites of hickory shuckworm and pecan nut casebearer with five new host-parasite records. Southwest Entomol. 1988;13:107–11.

[37] Balduf WV. Bionomic notes on the hexapodous parasites of *Acrobasis rubrifasciella*. Ann Entomol Soc Am. 1968;61:463–76. doi: 10.1093/aesa/61.2.463

[38] Tillman PG, Cate JR. Six new hosts of *Bracon mellitor* (Hymenoptera: Braconidae), with a review of recorded hosts. Environ Entomol. 1989;18:328–33. doi: 10.1093/ee/18.2.328

[39] Scaramozzino PL, Giovanni FD, Loni A, Ricciardi R, Lucchi A. Updated list of the insect parasitoids (Insecta, Hymenoptera) associated with *Lobesia botrana* (Denis & Schiffermüller, 1775) (Lepidoptera, Tortricidae) in Italy. 2. Hymenoptera, Ichneumonidae, Anomaloninae and Campopleginae. Zookeys. 2018;(772):47–95. doi: 10.3897/zookeys.772.25288 30018508 PMC6045682

[40] Carroll MR, Kearby WH. Microlepidopterous oak leaftiers (Lepidoptera: Gelechioidea) in central Missouri. J Kans Entomol Soc. 1978;51:457–71.

[41] GBIF Ocurrence Download [cited 2024 Dec 21]. 2023. Available from: GBIF.org. doi: 10.15468/dl.smx8rb

[42] Neiswander RB. Oriental fruit moth investigations in Ohio. II Bull 569. Wooster, Ohio: Ohio Agricultural Experiment Station; 1936. Available from: https://kb.osu.edu/server/api/core/bitstreams/35f58fd2-d549-5081-a53d-6dffe50947b3/content

[43] Strickland EH. Additions to the List of Ichneumonoidea from Alberta. Can Entomol. 1952;84(4):118–22. doi: 10.4039/ent84118-4

[44] Schwarzfeld MD. Ichneumonidae (Hymenoptera) of the Canadian prairies ecozone: a review. In: Giberson DJ and Cárcamo HA Arthropods of Canadian Grasslands (Volume 4): Biodiversity and Systematics Part 2. University of Northern British Columbia; 2014. pp. 317–97.

[45] Thiery D, Xuéreb A, Villemant C, Sentenac G, Delbac L, Kuntzman P. Les parasites larvaires de tordeuses de vignobles: aperçu de quelques espèces présentes dans 3 régions viticoles françaises. Larval parasites of vineyard tortricids: a brief overview from 3 french vine growing areas. IOBC/wprs Bull. 2001;24:135–42.

[46] Thompson WR. A catalogue of the parasites and predators of insect pests. Ann Entomol Soc Am. 1945; 38: 303–304. doi:10.1093/aesa/38.2.303

[47] Ricciardi R, Benelli G, Di Giovanni F, Lucchi A. The European grape berry moth, *Eupoecilia ambiguella* (Lepidoptera: Tortricidae): Current knowledge and management challenges. J Crop Prot. 2024;180:106641. doi:10.1016/j.cropro.2024.106641

[48] Bărbuceanu D, Andriescu I. The parasitoid complex of *Eupoecilia ambiguella* (Lepidoptera: Tortricidae) in a vineyard of Southern Romania. Muzeul Olteniei Craiova Oltenia Studii úi comunicări ùtiinĠele Naturii. 2012;28:99–104.

[49] Janzon L-Å. Description of the egg and larva of *Euphranta connexa* (Fabricius) (Diptera: Tephritidae) and of the egg of its parasitoid *Scambus brevicornis* (Gravenhorst) (Hymenoptera: Ichneumonoidea). Insect Syst Evol. 1982;13(3):313–6. doi: 10.1163/187631282x00363

[50] Solbreck C. Ecology and biology of *Euphranta connexa* (Fabr.) (Diptera: Tephritidae) - a seed predator on *Vincetoxicum hirundinaria* Med. (Asclepiadaceae). Entomol Tidskr. 2000;121:23–30.

[51] Zijp JP, Blommers LHM. Survival mode between yearly reproduction periods, and reproductive biology of *Scambus pomorum* (Hymenoptera: Ichneumonidae Pimplinae), a parasitoid of the apple blossom weeevil *Anthonomus pomorum* (Coleoptera: Curculionidae). Entomol Gen. 2002; 26: 29–46. doi:10.1127/entom.gen/26/2002/29

