# Supplementary material for: Exploring natural allies: Survey and identification of larval parasitoids of the American grape berry moth, Paralobesia viteana (Lepidoptera: Tortricidae) in northwestern Pennsylvania
Source: PLoS One. 2025 May 19;20(5):e0317274. doi: 10.1371/journal.pone.0317274 (PMC12088014; doi:10.1371/journal.pone.0317274)
Supplement: S1 Table — (PDF) [file pone.0317274.s001.pdf]

S1 Table. Characteristics of the grape berry moth sampling sites.

Sampling site	Vineyard area	Length of the vineyard border	Adjacent wooded area
1	51,672.16 m^2	139.23 m^2	7,835.49 m^2
2	4,625.16 m^2	90.94 m^2	10,784.30 m^2
3	31,209.86 m^2	205.21 m^2	10,784.30 m^2
4	50,501.10 m^2	120.18 m^2	21,261 m^2
5	24,077.15 m^2	88.04 m^2	13,369.33 m^2
6	38,908.24 m^2	384.19 m^2	834.51 m^2
