# Supplementary material for: Exploring natural allies: Survey and identification of larval parasitoids of the American grape berry moth, Paralobesia viteana (Lepidoptera: Tortricidae) in northwestern Pennsylvania
Source: PLoS One. 2025 May 19;20(5):e0317274. doi: 10.1371/journal.pone.0317274 (PMC12088014; doi:10.1371/journal.pone.0317274)
Supplement: S2 Table — (PDF) [file pone.0317274.s002.pdf]

S2 Table. GBM larval parasitism in field conditions per sampling site throughout the 2023 growing season.

**Sampling date**	**Sampling site**	**Number of grapes sampled**	**Number of parasitoids**	**Number of unparasitized GBM stages**	**Parasitism (%)**
6/15/23	1	Wild grape blossoms	5	2	71.43
	2		1	1	50.00
	3		0	3	0.00
	4		1	6	14.29
	5		0	2	0.00
	6		1	1	50.00
7/11/23	1	100	0	38	0.00
	2	49	0	22	0.00
	3	100	1	31	3.13
	4	100	4	53	7.02
	5	100	3	46	6.12
	6	100	0	50	0.00
7/24/23	1	100	4	48	7.69
	2	100	0	54	0.00
	3	100	0	28	0.00
	4	100	7	44	13.73
	5	100	9	69	11.54
	6	100	1	50	1.96
8/7/23	1	148	23	36	38.98
	2	150	11	68	13.92
	3	150	4	39	9.30
	4	150	1	21	4.55
	5	150	3	29	9.38
	6	150	8	28	22.22
8/23/23	1	100	3	30	9.09
	2	100	2	24	7.69
	3	108	2	46	4.16
	4	100	3	18	14.28
	5	100	3	34	8.11
	6	103	3	71	4.05
9/4/23	1	99	1	29	3.33
	2	98	0	23	0.00
	3	103	2	52	3.70
	4	100	1	46	2.13
	5	100	3	59	4.84
	6	100	3	79	3.65
