# Supplementary material for: Exploring natural allies: Survey and identification of larval parasitoids of the American grape berry moth, Paralobesia viteana (Lepidoptera: Tortricidae) in northwestern Pennsylvania
Source: PLoS One. 2025 May 19;20(5):e0317274. doi: 10.1371/journal.pone.0317274 (PMC12088014; doi:10.1371/journal.pone.0317274)
Supplement: S3 Table — (PDF) [file pone.0317274.s003.pdf]

S3 Table. GBM larval parasitism in field conditions per sampling site throughout the 2024 growing season.

Sampling date	Sampling site	Number of grapes sampled	Number of parasitoids	Number of unparasitized GBM stages	Parasitism (%)
6/24/24	1	100	0	53	0.00
	2	100	3	44	6.38
	3	100	0	39	0.00
	4	100	0	29	0.00
	5	100	1	59	1.67
	6	100	1	64	1.54
7/9/24	1	100	3	48	5.88
	2	100	8	41	16.33
	3	100	0	36	0.00
	4	100	3	60	4.76
	5	100	2	58	3.33
	6	100	0	51	0.00
7/23/24	1	100	10	19	34.48
	2	100	5	40	11.11
	3	100	5	33	13.16
	4	100	1	45	2.17
	5	100	3	25	10.71
	6	100	3	12	20.00
8/5/24	1	150	24	22	52.17
	2	150	2	98	2.00
	3	150	6	61	8.96
	4	150	15	35	30.00
	5	150	1	50	1.96
	6	150	3	66	4.35
8/20/24	1	150	8	42	16.00
	2	150	1	94	1.05
	3	150	9	66	12.00
	4	150	6	49	10.91
	5	150	0	83	0.00
	6	150	0	105	0.00
9/2/24	1	150	7	78	8.24
	2	150	17	134	11.26
	3	150	7	44	13.73
	4	150	4	57	6.56
	5	150	0	81	0.00
	6	150	1	97	1.02
